# The Sirt1 Activator SRT1720 Mitigates Human Monocyte Activation and Improves Outcome During Gram-Negative Pneumosepsis in Mice

**DOI:** 10.3390/ijms26199309

**Published:** 2025-09-24

**Authors:** Mathieu Blot, Valentine Léopold, Regina de Beer, Sandrine Florquin, Joe M. Butler, Cornelis van’t Veer, Alex F. de Vos, Tom van der Poll

**Affiliations:** 1 Center of Infection & Molecular Medicine, Amsterdam University Medical Center, Location Academic Medical Center, University of Amsterdam, 1105 AZ Amsterdam, The Netherlandsa.f.devos@amsterdamumc.nl (A.F.d.V.); t.vanderpoll@amsterdamumc.nl (T.v.d.P.); 2 Amsterdam Infection & Immunity Institute, Amsterdam University Medical Center, University of Amsterdam, 1105 AZ Amsterdam, The Netherlands; 3 Département de Maladies Infectieuse, Hôpital Universitaire Dijon-Bourgogne, 21079 Dijon, France; 4 Labex LipSTIC, LNC UMR1231, INSERM, Université Bourgogne-Europe, 21078 Dijon, France; 5 Department of Pathology, Amsterdam University Medical Center, University of Amsterdam, 1105 AZ Amsterdam, The Netherlands; 6 Division of Infectious Diseases, Amsterdam University Medical Center, University of Amsterdam, 1105 AZ Amsterdam, The Netherlands

**Keywords:** Sirt1, SRT1720, pneumonia, *Klebsiella pneumoniae*, lipopolysaccharide, monocyte activation, immune response

## Abstract

Community-acquired pneumonia (CAP) is a leading cause of death, with mortality linked to an unbalanced host response. Sirtuin (Sirt)1, a histone deacetylase, regulating metabolism and epigenetics, may be fundamental in activating the innate immune response. Sirt1 mRNA expression was significantly reduced in monocytes from CAP patients (*n* = 76) upon admission compared to healthy controls (*n* = 42), with levels returning to normal after 30 days. Pharmacological activation of Sirt1 with SRT1720 decreased LPS- and *K. pneumoniae*-induced IL-6 release in primary human monocytes and decreased NF-κB activation in THP1 cells. In a mouse *K. pneumoniae* pneumosepsis model, SRT1720 strongly reduced neutrophil influx and degranulation markers in bronchoalveolar lavage fluid, lowered pulmonary concentrations of IL-6 and TNF-α, and reduced lung pathology scores. Simultaneously, it reduced neutrophil content in liver tissue and plasma transaminase levels, alongside a trend toward reduced liver necrosis. Plasma IL-6 and TNF-α were significantly lower in SRT1720-treated mice at 42 h. Finally, while SRT1720 did not impact bacterial loads in the lungs, it reduced bacterial burden in blood, with a similar trend observed in liver homogenates. In conclusion, the Sirt1 activator SRT1720 exerts anti-inflammatory effects on human monocytes, reduces local and systemic inflammation and organ injury, and diminishes bacterial dissemination in murine pneumosepsis.

## 1. Introduction

Pneumonia remains a leading cause of death worldwide and is the main cause of sepsis and septic shock [1]. During the early stages of the host response to infection, myeloid immune cells have critical requirements, most notably energy, pathogen killing capacity and resistance to apoptosis to promote pathogen elimination [2,3]. They achieve these needs by shifting from oxidative phosphorylation to aerobic glycolysis, a change in energy metabolism known as the Warburg effect [2]. Studies have identified a crucial role of cellular metabolism in the functional fate of immune cells, opening up new therapeutic avenues [2,4].

Sirtuin (Sirt)1, a highly conserved NAD+-dependent histone deacetylase belonging to the sirtuin family, plays a central role in regulating cellular and energy metabolism, inflammation and oxidative stress. Sirt1 represses transcriptomic activation and controls p53-mediated apoptosis through its function as an epigenetic regulator [5,6]. By acetylating histone proteins, Sirt1 facilitates chromatin compaction and gene silencing, thereby exerting anti-inflammatory properties [5,7]. In vitro, studies show that the deletion of SIRT1 in myeloid cells results in increased acetylation of nuclear factor-kappa B (NF-κB), which enhances its transcriptional activity and promotes the expression of pro-inflammatory genes [8,9]. Conversely, the overexpression of Sirt1 during bone-marrow-derived macrophage differentiation increased their proliferative capacity both in vitro and in vivo [10], while also reducing LPS-induced macrophage apoptosis [11]. Sirt1 promotes the shift in macrophages from pro-inflammatory M1 to anti-inflammatory M2 by reducing M1 markers such as TLR4, p-NF-kB, IL-1β, and iNOS and increasing M2 markers like Arg1, thus helping to mitigate inflammation [12,13].

Evidence suggest a beneficial role of Sirt1 activation in the context of sepsis [7,13]. Sirt1 is downregulated in macrophages from mouse models of sepsis or upon LPS stimulation [14]. In a mouse model of endotoxemia, pharmacological activation of SIRT1 by SRT1720 reduced macrophage apoptosis and lung inflammation [11]. Additionally, in a rat model of peritonitis, resveratrol, another SIRT1 activator, provided protection against sepsis-induced liver injury [15]. In contrast, Sirt1 deletion exacerbated systemic inflammation and worsened acute kidney injury and mortality in mice [16,17]. Finally, the small molecule SRT2104, a Sirt1 activator, has been shown to attenuate lipopolysaccharide-induced release of inflammatory cytokines in healthy volunteers [18].

However, there is little data on the regulation of Sirt1 in myeloid cells during pneumonia. Recent findings suggest that pneumonia induces a functional knockdown of Sirt1 in the lungs of wild-type mice [19]. In light of these data, the pharmacological activation of Sirt1 could potentially be beneficial for resolving inflammation and improving outcomes during pneumosepsis.

We hypothesized that Sirt1 plays a critical role in innate immune activation and that Sirt1 activation could improve outcomes in bacterial pneumosepsis. To this end, we first performed transcriptomic analysis of blood monocytes from patients with community-acquired pneumonia (CAP). Next, we investigated the effect of the small molecule SRT1720, a Sirt1 activator, in human monocytes, as well as in a mouse model of pneumonia.

## 2. Results

### 2.1. Sirt1 mRNA Expression Is Decreased in Monocytes of CAP Patients

**Figure 1:**
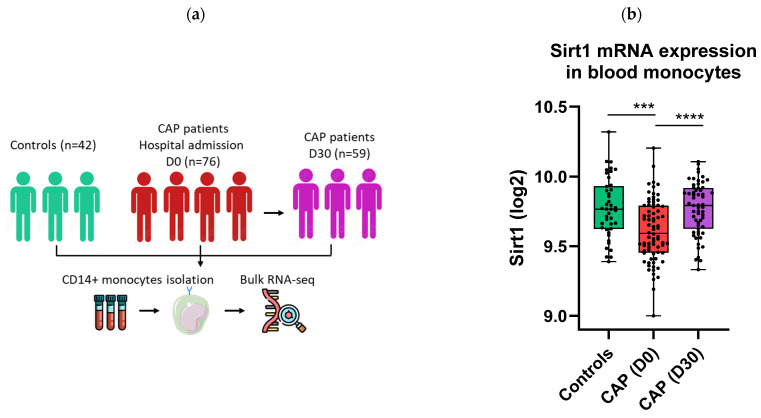
Sirt1 mRNA expression is reduced in monocytes of CAP patients. RNA sequencing data of blood CD14+ monocytes from CAP patients (at admission and 30 days later) and healthy controls were obtained from previously published data (accession number GSE160329) (**a**). Boxplot showing the expression of Sirt1 mRNA in monocytes of CAP patients at admission (D0), 30 days later (D30), and in healthy controls (**b**). Each dot represents either a healthy control or a patient. Healthy controls are shown in green, patients with pneumonia at admission in red, and patients at day 30 in purple. Differences were analyzed using the Kruskal–Wallis analysis of variance test and Mann–Whitney U test followed by False Discovery Rate corrected multiple comparison. *** *p* < 0.001, **** *p* < 0.0001.

It has been previously shown that Sirt1 is downregulated in macrophages in preclinical models of sepsis [14] and may play a role in orchestrating innate immune activation, particularly through the activation of NF-κB and the subsequent production of pro-inflammatory cytokines such as IL-6 and TNF-α [7,9]. We first investigated how Sirt1 was regulated in circulating monocytes of CAP patients. For this purpose, blood CD14+ monocytes were isolated from CAP patients within 24 h after hospitalization admission (*n* = 76, acute stage) and 30 days thereafter (*n* = 59, recovery stage), as well as from 42 control participants (Figure 1a). Sirt1 mRNA expression in monocytes harvested from CAP patients at admission was significantly decreased, when compared with Sirt1 mRNA expression in monocytes from healthy controls, with a return to normal levels 30 days later (Figure 1b).

### 2.2. The Sirt1 Activator SRT1720 Inhibits LPS and K. Pneumoniae-Induced Monocyte Activation

**Figure 2:**
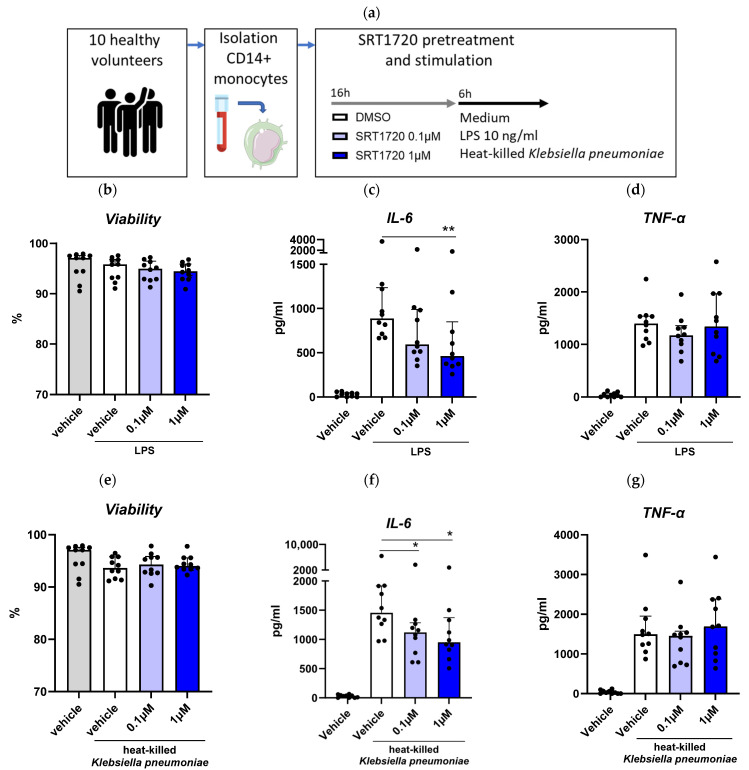
The Sirt1 activator SRT1720 dampens LPS and *Klebsiella pneumoniae*-induced monocyte activation and metabolism. Blood CD14+ monocytes were isolated from 10 healthy volunteers and treated with 0.1 or 1 μM SRT1720 or DMSO (vehicle control) before stimulation with 10 ng/mL LPS or heat-killed *Klebsiella pneumonia* for 6 h (**a**). Cell viability (**b**), concentrations of IL-6 (**c**), and TNF-α (**d**) in supernatant were measured after LPS stimulation. Cell viability (**e**), concentrations of IL-6 (**f**), and TNF-α (**g**) in supernatant were measured after *Klebsiella pneumoniae* stimulation. Bar graphs represent medians (interquartile ranges) of the mean values of technical replicates (*n* = 4) of each healthy volunteer. Gray bars indicate vehicle-pretreated unstimulated monocytes, white bars vehicle-pretreated stimulated monocytes, light blue bars SRT1720-pretreated (0.1 µM) stimulated monocytes, and dark blue bars SRT1720-pretreated (1 µM) stimulated monocytes. Comparisons between SRT1720 concentrations and DMSO were determined using the Friedman test for paired data with Dunn’s correction for multiple comparisons. * *p* < 0.05, ** *p* < 0.01.

We aimed to determine the effect of a small molecule activator of Sirt1, namely SRT1720 [20,21], on monocytes activation from samples of ten healthy volunteers (Figure 2a). SRT1720 did not affect cell viability (Figure 2b). Pretreatment with SRT1720 reduced LPS-induced IL-6 release (at 0.1 and 1 μM), with no significant impact on TNF-α release (Figure 2c,d). In monocyte cultures stimulated with heat-killed *K. pneumoniae*, SRT1720 decreased IL-6 release (at both 0.1 and 1 μM) without altering TNF-α levels (Figure 2e–g). 

### 2.3. SRT1720 Reduces NF-κB Activation in THP1 Monocytes

**Figure 3:**
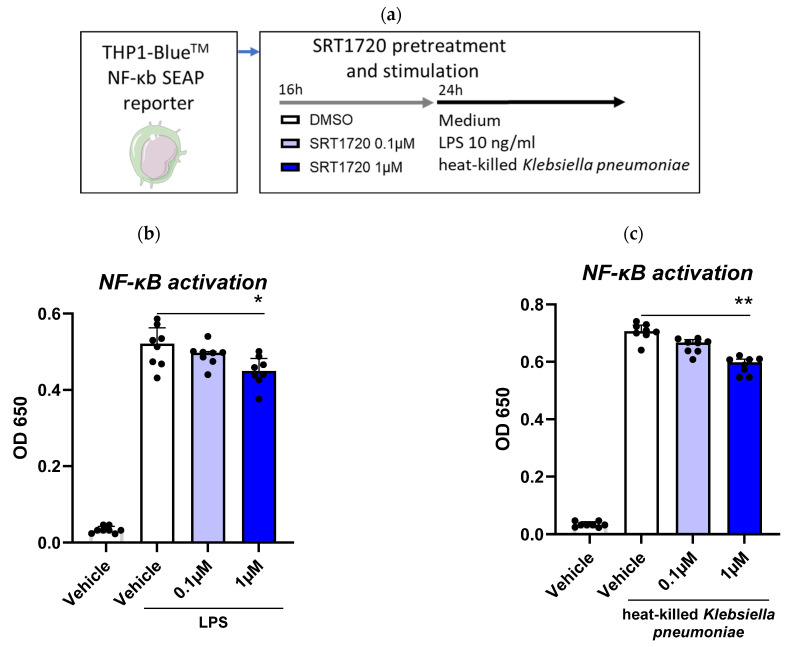
SRT1720 reduces NF-κB activation in THP1 monocytes. XBlue cells were treated with 0.1 or 1 μM SRT1720 or DMSO (vehicle control) for 16 h before stimulation with 10 ng/mL LPS or heat-killed *Klebsiella pneumonia* for 24 h (**a**). SEAP activity, used as a proxy for NF-κB activation, was measured in THP1-XBlue cells and expressed as OD650. Bar graphs represent the medians (interquartile ranges) of 8 technical replicates from a representative experiment out of four independent experiments conducted with LPS stimulation (**b**) and *Klebsiella pneumoniae* stimulation (**c**). Comparisons between SRT1720 concentrations and DMSO were determined using the Friedman test for paired data with Dunn’s correction for multiple comparisons: * *p* < 0.05, ** *p* < 0.01.

As Sirt1 is a well-known regulator of NF-κB [9,13], we used THP1 X-blue cells with a NF-κB SEAP reporter to investigate the effect of SRT1720 on NF-κB activation in monocytic cells (Figure 3a). We observed that pretreatment of THP1 X-blue cells with SRT1720 significantly decreased LPS and *K. pneumoniae*-induced NF-κB activation in a dose dependent manner (Figure 3b,c).

### 2.4. SRT1720 Reduces Bacterial Dissemination During in Vivo Klebsiella Pneumosepsis

**Figure 4:**
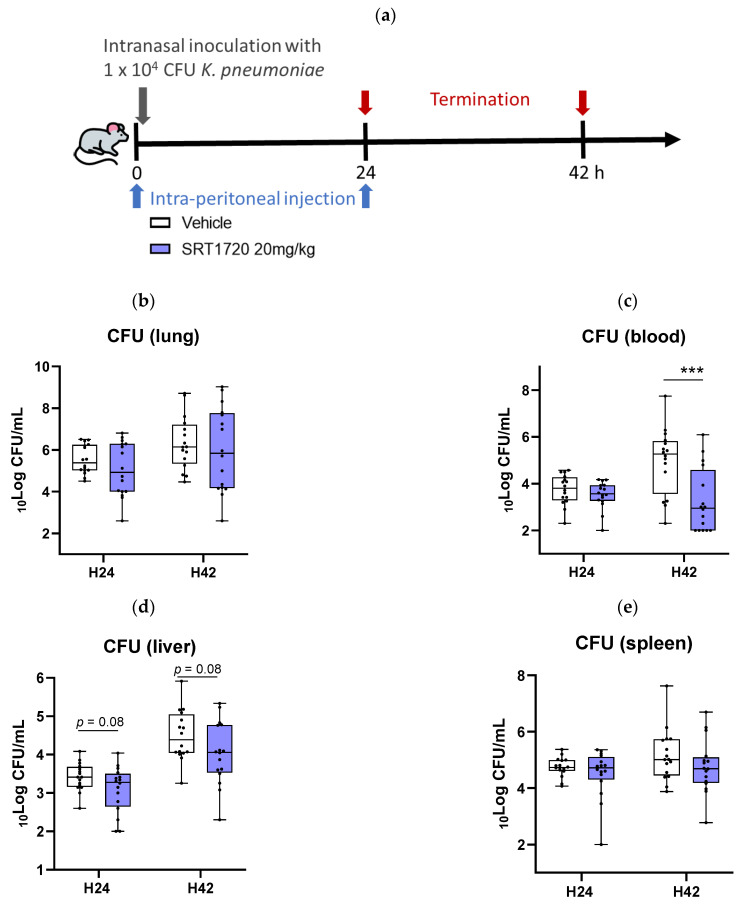
SRT1720 reduces bacterial dissemination during *Klebsiella* pneumosepsis. Mice were injected intraperitoneally with 20 mg/kg SRT1720 or DMSO (vehicle) before and 24 h after intranasal inoculation with approximately 104 colony-forming units (CFUs) of *Klebsiella pneumoniae* and euthanized 24 or 42 h post-inoculation (**a**). Bacterial loads (CFUs per milliliter) were determined in the lung (**b**), blood (**c**), liver (**d**) and spleen (**e**). Bacterial load comparisons between SRT1720- and DMSO-treated mice (*n* = 16/group, from 2 independent experiments) were performed at both time points using the Mann–Whitney test with False Discovery Rate correction for multiple comparisons: *** *p* < 0.001.

To understand the effect of SRT1720 on host defense during pneumonia-derived sepsis, mice were injected intraperitoneally with SRT1720 (20 mg/kg) [11] or vehicle immediately after and 24 h after infection with a virulent strain of *K. pneumoniae* (Figure 4a). Mice were euthanized at 24 or 42 h post-infection to assess bacterial loads and inflammatory responses. SRT1720 did not influence bacterial loads at the primary infection site (i.e., the lungs) at either time point (Figure 4b). At the late time point (42 h) SRT1720 treated mice had lower bacterial burdens in blood (Figure 4c, *p* = 0.0009 versus vehicle control); a similar trend was seen in liver homogenates (Figure 4d, *p* = 0.08). SRT1720 did not alter bacterial numbers in the spleen (Figure 4e).

### 2.5. SRT1720 Mitigates Lung Inflammation and Pathology During Klebsiella Pneumosepsis

**Figure 5:**
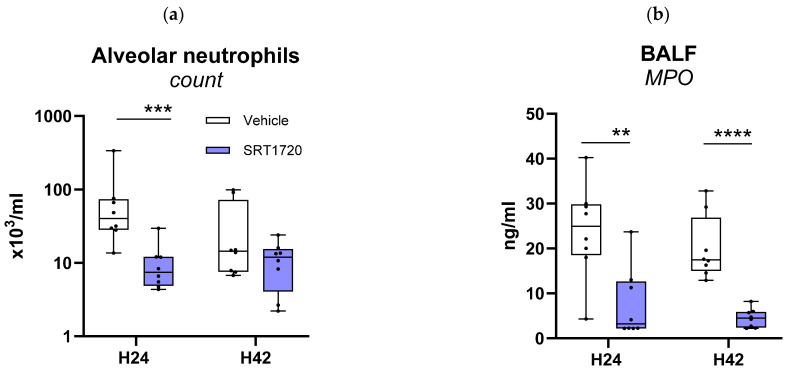

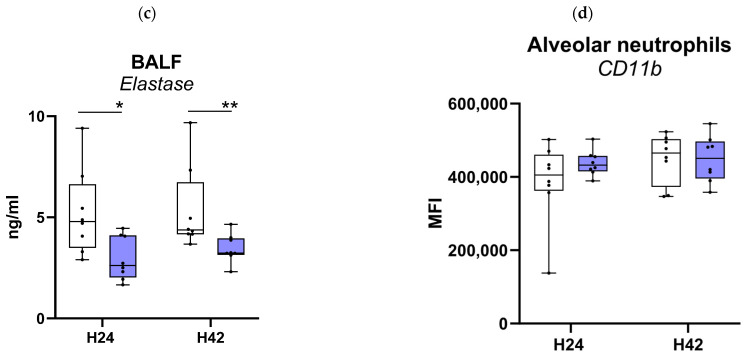
SRT1720 mitigates alveolar inflammation during *Klebsiella* pneumosepsis. Mice were injected intraperitoneally with 20 mg/kg SRT1720 or DMSO (vehicle) before and 24 h after intranasal inoculation with approximately 104 colony-forming units (CFUs) of *Klebsiella pneumoniae* and euthanized 24 or 42 h post-inoculation. Bronchoalveolar lavage fluid (BALF) was collected immediately after sacrifice in one experiment (*n* = 8/group). Neutrophils count (via flow cytometry) (**a**), concentrations of myeloperoxidase (MPO) (**b**) and elastase (**c**) in BALF, and CD11b expression on alveolar neutrophils (**d**). Comparisons between SRT1720- and DMSO-treated mice were performed at both time points using the Mann–Whitney test with False Discovery Rate correction for multiple comparisons. * *p* < 0.05, ** *p* < 0.01, *** *p* < 0.001, **** *p* < 0.0001.

**Figure A1:**
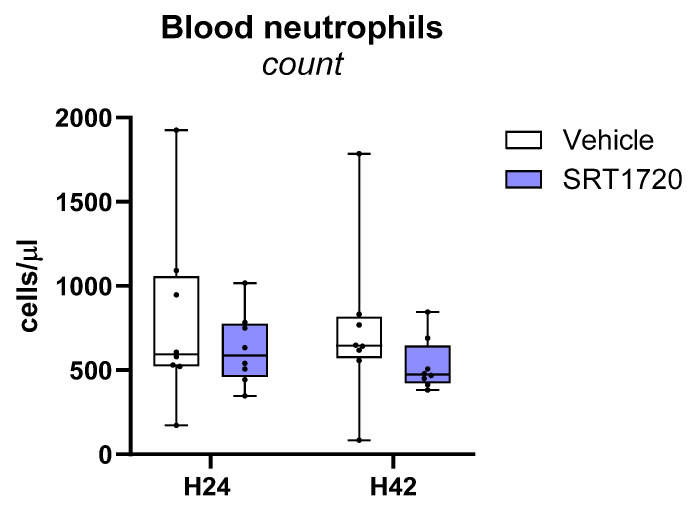
SRT1720 has no effect on blood neutrophils count. Mice were injected intraperitoneally with 20 mg/kg SRT1720 or DMSO (vehicle) before and 24 h after intranasal inoculation with approximately 104 colony-forming units (CFUs) of Klebsiella pneumoniae and were euthanized 24 and 42 h post-inoculation. Blood neutrophil count was measured with flow cytometry. Comparisons between SRT1720- and DMSO-treated mice were performed at both time points (*n* = 8/group) using the Mann–Whitney test with False Discovery Rate correction for multiple comparisons.

To obtain an initial understanding of how SRT1720 affects the inflammatory response in the airways during *Klebsiella*-induced pneumonia, we measured cytokines (IL-6, TNF-α) and chemokines (CXCL1 and CXCL2) in BALF obtained 24 or 42 h after infection (Table 1). SRT1720 strongly reduced the alveolar concentrations of all these mediators at 24 h, an effect that was partially detectable after 42 h (Table 1). SRT1720 decreased neutrophil influx into BALF, significantly so at 24 h (Figure 5a), which was corroborated by lower levels of cell-free MPO and elastase in BALF (Figure 5b,c). Neutrophil counts in peripheral blood were not influenced by SRT1720 (Figure A1), suggestive of a specific effect on neutrophil recruitment during pneumonia. SRT1720 did not alter the activation state of neutrophils in BALF, as indicated by similar neutrophil CD11b expression (Figure 5d).

**Figure 6:**
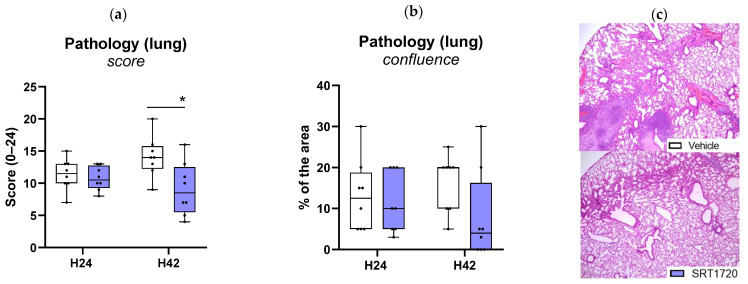
SRT1720 reduces lung pathology during *Klebsiella* pneumosepsis. Mice were injected intraperitoneally with 20 mg/kg SRT1720 or DMSO (vehicle) before and 24 h after intranasal inoculation with approximately 10^4^ colony-forming units (CFUs) of *Klebsiella pneumoniae* and euthanized 24 or 42 h post-inoculation. The extent of lung inflammation scored on hematoxylin and eosin-stained tissue sections shown as total pathology score (**a**), and confluence (**b**,**c**) (*n* = 8/group). Comparisons between SRT1720- and DMSO-treated mice were performed at both time points using the Mann–Whitney test with False Discovery Rate correction for multiple comparisons. * *p* < 0.05.

To determine the impact of SRT1720 on lung pathology, H&E stained lung sections were semi-quantitatively scored with reference to histological features characteristic of pneumonia [22]. As reported previously, *K. pneumoniae*-induced pneumonia was associated with gross lung pathology [23,24]. SRT1720 reduced total pathology scores at 42 h, while confluent lung inflammation was not different between SRT1720-treated and vehicle-treated mice (Figure 6a,c).

### 2.6. SRT1720 Mitigates Distant Organ Injury During Klebsiella Pneumosepsis

**Figure 7:**
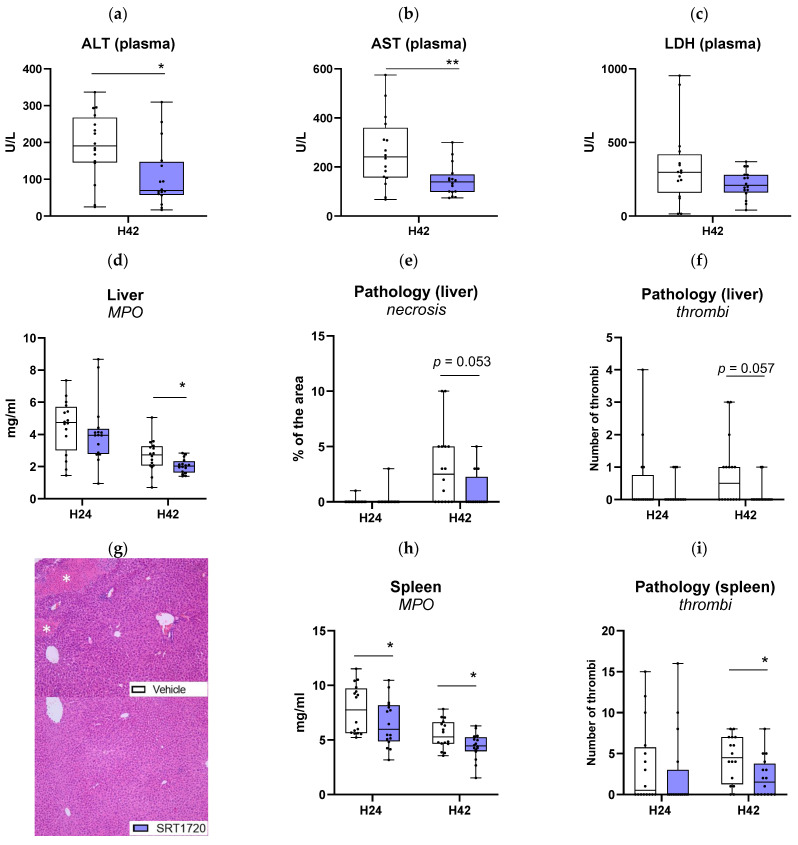
SRT1720 dampens distant organs injury during *Klebsiella* pneumosepsis. Mice were injected intraperitoneally with 20 mg/kg SRT1720 or DMSO (vehicle) before and 24 h after intranasal inoculation with approximately 10^4^ colony-forming units (CFUs) of *Klebsiella pneumoniae* and euthanized 24 or 42 h post-inoculation. Plasma levels of alanine transaminase (ALT; (**a**)), aspartate transaminase (AST; (**b**)), lactate dehydrogenase (LDH; (**c**)). Myeloperoxidase concentrations in liver homogenates (**d**). The extent of liver necrosis (**e**) and thrombi formation (**f**) on hematoxylin and eosin-stained tissue sections (**g**). Myeloperoxidase concentrations in spleen homogenates (**h**), and thrombi formation in eosin-stained spleen sections (**i**). Comparisons between SRT1720- and DMSO-treated mice were performed at both time points (*n* = 16/group) using the Mann–Whitney test with False Discovery Rate correction for multiple comparisons. * *p* < 0.05, ** *p* < 0.01.

This model of *Klebsiella*-induced pneumosepsis is associated with the development of distant organ damage at later stages after infection, particularly reflected by rises in the plasma concentrations of liver enzymes AST and ALT, and the general cell injury marker LDH. SRT1720 significantly reduced plasma AST and ALT levels at 42 h after infection, while not affecting plasma LDH levels (Figure 7a–c). Consistent with an anti-inflammatory effect SRT1720 treatment was associated with a lower neutrophil content in liver tissue, as indicated by reduced MPO levels in liver homogenates (Figure 7d). Furthermore, examination of liver tissue slides revealed a trend toward reduced liver necrosis (*p* = 0.053 versus vehicle controls; Figure 7e–g) and thrombus formation (*p* = 0.057; Figure 7f). In agreement, spleen tissue also showed lower MPO levels (Figure 7h) and less thrombus formation (Figure 7i) in SRT1720-treated mice. SRT1720 reduced plasma IL-6 and TNF-α levels at 42 h after infection, while not affecting plasma IL-12 or IFN-γ (Table 2).

## 3. Discussion

Here we report that Sirt1 expression is reduced in blood monocytes of patients with CAP, normalizing by day 30 after admission. We further demonstrate that activating Sirt1 with the small molecule SRT1720 lowers the activation of primary human monocytes induced by LPS and *Klebsiella pneumoniae* in vitro. Additionally, we demonstrate that treatment with SRT1720 mitigates the inflammatory response in a mouse model of *Klebsiella* pneumosepsis, reducing lung and distant organ damage and the dissemination of the infection.

In pneumosepsis, myeloid cell activation is a central driver of organ damage and can lead to death if the inflammatory response becomes excessive [1]. Notably, monocytes and macrophages require shifts in energy metabolism and resistance to apoptosis to effectively promote pathogen elimination. These processes are regulated by cellular metabolic pathways, including the well-documented Warburg effect [2]. Given that Sirt1 is a key regulator of cellular metabolism and immune function, its modulation presents an attractive strategy in the context of pneumosepsis. This study highlights the potential of Sirt1 activation, specifically through SRT1720, as a therapeutic approach for bacterial pneumosepsis, aimed at controlling myeloid cell hyperactivation.

Our results first demonstrate a significant reduction in Sirt1 mRNA expression in monocytes from patients with CAP, particularly during the acute phase of the infection. This finding is consistent with previous studies in preclinical sepsis models, where Sirt1 downregulation was associated with heightened inflammation and poor outcomes [14]. Additionally, pneumonia has been shown to induce a functional knockdown of Sirt1 in the lungs of mice within four hours, leading to Sirt1 levels comparable to those observed in Sirt1-deficient mice [19], indicating that this effect is not limited to the systemic compartment. The normalization of Sirt1 expression during the recovery phase in CAP patients suggests that Sirt1 may play a role in regulating the resolution of the inflammatory response following pneumonia.

Secondly, we observed that the small compound SRT1720, a Sirt1 activator, dampened the activation of human monocytes induced by LPS and *Klebsiella pneumoniae*, as evidenced by decreased IL-6 release. This aligns with the established role of Sirt1 in modulating inflammation by deacetylating and inhibiting NF-κB, a key transcription factor involved in the expression of various pro-inflammatory cytokines, chemokines, and adhesion molecules [9]. Using THP1-XBlue NF-κB reporter monocytic cells, we confirmed that SRT1720 inhibits NF-κB activation in a dose-dependent manner. These findings highlight the role of Sirt1 in modulating critical inflammatory pathways and underscore the potential of SRT1720 in reducing hyperinflammation, a key driver of organ injury in sepsis.

Using a *Klebsiella*-induced pneumosepsis model, we demonstrated that SRT1720 treatment reduced alveolar inflammation within 24 h and systemic inflammation by 42 h, confirming its anti-inflammatory effects in vivo. Importantly, SRT1720-treated mice exhibited reduced liver injury, indicated by lower AST and ALT levels, and decreased liver necrosis and thrombus formation, reflecting its protective effects against distant organ damage. As a stress sensor, Sirt1 helps maintain cellular homeostasis and survival by deacetylating regulatory proteins and modulating various transcription factors, steering the cell toward a cytoprotective pathway [25]. These effects are mediated through reduced apoptosis via deacetylation of p53, regulation of mitochondrial biogenesis, and decreased inflammation and oxidative stress [9,25,26]. By dissecting the mechanisms through which SRT1720 exerts its beneficial effects, we observed a decrease in neutrophil influx into BALF and reduced neutrophil degranulation, as evidenced by lower levels of cell-free MPO and elastase in the alveolar space. We also noted lower neutrophil content in liver and spleen tissue, as indicated by reduced MPO levels in homogenates.

Neutrophil influx is a double-edged sword in sepsis. While essential for host defense against infection, uncontrolled neutrophil activation and accumulation can lead to significant organ dysfunction and contribute to high mortality [27]. Neutrophil counts in peripheral blood were not influenced by SRT1720, suggesting a specific effect on neutrophil recruitment during pneumonia. Similarly, previous studies have shown that Sirt1 upregulation via antisense RNA diminished lung edema, epithelial cell apoptosis, neutrophil infiltration, and inflammatory responses in a mouse model of peritonitis [28]. By inhibiting NF-κB, SIRT1 reduces the activation of monocytes and macrophages, ultimately leading to decreased chemokine production. Our results showed that alveolar concentrations of CXCL1 and CXCL2 were indeed reduced in animals treated with SRT1720, as early as 24 h post-infection. Conversely, these data contradict findings by Labiner et al. who reported that Sirt1 deletion in mice was associated with decreased neutrophil infiltration in the lungs and a shift toward a more immature neutrophil phenotype [19]. In our study, SRT1720 did not alter the activation state of neutrophils in BALF, as indicated by similar CD11b expression.

Finally, while bacterial clearance in the lungs remained unaffected, SRT1720 significantly reduced bacterial burdens in the bloodstream and liver at later time points. This suggests that SRT1720 does not impair immune defense at the primary site of infection but instead limits systemic infection spread. Several hypotheses can be proposed, such as reduced pulmonary-to-systemic bacterial translocation, as pulmonary damage appeared to be limited in animals treated with SRT1720. Previous research indicated that SRT1720 improved lung function, including airway resistance and pulmonary dynamic compliance, in rats with emphysema by inhibiting type II alveolar epithelial cell apoptosis [29]. Moreover, in mouse models of LPS-induced sepsis, SRT1720 significantly reduced LPS-induced lung injury by limiting hyperpermeability through the reduction in reactive oxygen species production and SIRT1-dependent suppression of NF-κB–mediated inflammation with preservation of tight junction proteins, thereby reducing endothelial permeability [30,31], and by reducing LPS-induced macrophage apoptosis through the inhibition of the endoplasmic reticulum stress response [11].

From a therapeutic perspective, our findings suggest that pharmacological activation of Sirt1 could be a promising strategy to improve outcomes in bacterial pneumosepsis. SRT1720 could potentially attenuate the dysregulated immune response that characterizes sepsis, thereby reducing organ damage and improving survival. This is particularly relevant in sepsis, where hyperinflammation often leads to widespread tissue injury and multiple organ failure [32].

Despite the promising findings, several limitations should be considered. First, our study focused on the acute phase of pneumonia and sepsis; the long-term effects of Sirt1 activation on immune function and pathogen clearance were not explored. In this model studies on the consequence of SRT1720 treatment on long-term sepsis sequelae, such as immunosuppression or persistent organ dysfunction, cannot be assessed, since mice are expected to die shortly after the latest sampling time point (42 h) [24]. Additionally, we did not investigate the effect of SRT1720 on survival because according to Dutch law animal welfare restrictions apply to mouse studies with mortality as endpoint. Second, the specificity of SRT1720’s action on Sirt1 requires further validation, as contradictory results have been observed in the literature depending on the approach used to modulate Sirt1, and some studies have indicated that Sirt1 activators may have off-target effects [33]. Hence, the effects of SRT1720 described here cannot be definitely attributed to Sirt1 activation. For this study, using Sirt1-deficient mice is required. Thus, the mechanistic understanding of SRT1720’s effects warrants further investigation, particularly regarding its potential to modulate monocyte/macrophage responses and neutrophil recruitment. Although we demonstrated that SRT1720 reduced NF-κB activation, direct biochemical evidence of SIRT1 enzymatic inhibition and proximal cellular events was not provided, and potential off-target effects of the compound cannot be excluded. In addition, the effect of SRT1720 on other monocyte activation or immunosuppression markers, such as HLA-DR, CD80/CD86, CD38 or PD-L1 [34], was not assessed. Similarly, we did not analyze the impact of SRT1720 on distinct monocyte subsets (classical, intermediate, non-classical), which could display differential responses. Other innate immune populations, such as dendritic cells, γδ T cells, or innate lymphoid cells (ILC1/ILC3), were not evaluated either, even though they play important roles in shaping lung inflammation and could contribute to the reduced neutrophil recruitment observed. Moreover, we did not assess the effect of SRT1720 on additional pro-inflammatory cytokines (e.g., IL-8 or IFN-γ). Likewise, epithelial-derived alarmins such as IL-1α or IL-33 were not measured, even though they may influence neutrophil-attracting chemokines and overall lung inflammation.

Moreover, the possible action of SRT1720 on endothelial cells was not investigated in our experimental setting, and we did not assess circulating markers of endothelial activation (e.g., sICAM-1, sVCAM-1) or integrity (e.g., soluble VE-cadherin, angiopoietin-2). Previous studies have suggested that SIRT1 activation may reduce endothelial permeability and ROS production in models of LPS-induced injury, but this requires confirmation in pneumonia and sepsis. Another limitation relates to the use of a single inbred strain (C57BL/6 mice), which restricts the generalizability of our results given the genetic diversity of human populations; future studies in other mouse strains or larger animal models (such as rabbits) will be required to strengthen translational relevance.

Lastly, while informative, the use of preclinical models may not fully capture the complexity of human sepsis, necessitating further clinical studies to validate the therapeutic potential of SRT1720 in patients.

## 4. Materials and Methods

### 4.1. Study Population

Monocyte Sirt1 mRNA levels in CAP patients and healthy controls included in a longitudinal, observational cohort study, were obtained from previously published RNA sequencing data from our group, publicly available in the Gene Expression Omnibus of the National Center for Biotechnology Information with accession number GSE160329 [35]. For detailed information on inclusion criteria and methods, please refer to reference [35].

Additionally, for in vitro analysis, we included 10 healthy controls, all hospital employees with no signs of infectious diseases. Informed consent was obtained from all participants.

### 4.2. Ex Vivo-Experiments Using Monocytes from Healthy Controls

Heparinized blood was diluted 1:1 with phosphate-buffered saline (PBS). Peripheral blood mononuclear cells (PBMCs) were isolated using density-gradient centrifugation with Ficoll-Paque PLUS (GE Healthcare, Chicago, IL, USA). CD14+ monocytes were then purified by positive selection using MACS CD14 microbeads, following the manufacturer’s instructions (Miltenyi Biotec, Bergisch Gladbach, Germany). Monocyte purity, assessed by flow cytometry, consistently exceeded 90%.

For ex vivo stimulations, purified monocytes were seeded into a cell-repellent surface 96-well plate (1.5 × 10^5^ cells per well) and incubated for 24 h at 37 °C with 5% CO_2_ and 95% humidity in a total volume of 150 μL Roswell Park Memorial Institute (RPMI) medium (GIBCO, 31870-025) supplemented with 10% sterile fetal calf serum, 200 mM glutamax (Thermo Fisher, Waltham, MA, USA; 35050-038), 1 mM pyruvate (Thermo Fisher, Waltham, MA, USA; 11360-039), 20 mM HEPES (Gibco, Thermo Fisher, Waltham, MA, USA; 15630-056), and 20 mg/mL gentamycin (Lonza, Basel, Switzerland; 17-519Z). The cells were treated with 0.2% DMSO (vehicle), 0.1 or 1μM SRT1720 [20,21] (Selleckchem, Houston, TX, USA) for 16 h. After incubation, plates were centrifuged and the supernatant discarded. Cells were then stimulated with 10 ng/mL lipopolysaccharide (LPS, from *Escherichia coli* 055:B5 Ultrapure, Invivogen, Toulouse, France), heat-killed *Klebsiella pneumoniae* (American Type Culture Collection 43816) (MOI = 10), or complete medium for 6 h. Supernatants were stored at −20 °C until analysis within 2 weeks.

### 4.3. Measurement of NF-κB Activity

Human THP-1-XBlue^TM^ NF-kB cells (Invivogen, thp-nfkbv2) were cultured in RPMI 1640 medium supplemented with 10% sterile fetal calf serum, 2 mM L-glutamine, 50 U/mL Normocin, 100 U/mL penicillin and 100 µg/mL streptomycin, 1 mM pyruvate (Thermo Fisher, 11360-039), 20 mM HEPES (Gibco, Thermo Fisher, Waltham, MA, USA; 15630-056), 1.5 g/L sodium bicarbonate, and 50 µM 2-Mercaptoethanol. THP-1 XBlue cells contain a reporter construct expressing secreted alkaline phosphatase (SEAP) to assess activation of the NF-κB transcription factor. Upon stimulation with 10 ng/mL LPS for 24 h at 37 °C, NF-κB activation occurs, promoting SEAP secretion. SEAP levels were detected in the culture medium by incubating supernatants with Quanti-Blue medium (InvivoGen, San Diego, CA, USA) for 4 h, followed by quantification at 650 nm using an ELISA reader (Titertek Instruments, Huntsville, AL, USA).

### 4.4. Mice

Pathogen free 8- to 10-week-old female C57BL/6 mice were purchased from Charles River (Leiden, The Netherlands). All animals were specific-pathogen-free and housed in the Animal Research Institute Amsterdam facility under standard care conditions. All experiments were carried out in accordance with the Dutch Experiments on Animals Act and were approved by the local animal welfare committee of the Academic Medical Center (protocol DIX17-4125-1-89, approved on January 28, 2021).

### 4.5. Experimental Study Design

Experimental pneumonia was induced as previously described [22,23,36]. This model is associated with dissemination of the infection from the lungs to distant organs, with systemic inflammatory responses, organ injury, and eventually death [23,24]. In short, a virulent strain of *K. pneumoniae* serotype 2 (43816; ATCC, Rockville, MD, USA) was grown in TSB medium to log phase. Cell suspensions were washed and diluted in isotonic saline. Mice were anesthetized by inhaling isoflurane carried in oxygen and thereafter 50 μL of a suspension containing 1 × 10^4^ colony-forming units (CFU) of *K. pneumoniae* was inoculated intranasally. Just prior intranasal inoculation, mice were injected intraperitoneally with either 20 mg/kg body weight of SRT1720 at a dilution of 2,5 mg/mL or vehicle (10% DMSO in normal saline) [11]. Animals were sacrificed at 24 or 42 h after induction of pneumonia. Those sacrificed at 42 h, were reinjected 24 h after the first injection. Each experiment was performed with 8 mice per group. The experiment was repeated and data pooled (*n* = 16/group) as indicated in the figure and table legends. Lungs for pathology and bronchoalveolar lavage fluid (BALF) were obtained in separate experiments to avoid dilution of samples (*n* = 8/group for both analyses) as described [36].

### 4.6. Bacterial Burden Determination

At the indicated time points, lungs, spleen, and liver were harvested and placed in sterile tubes. Blood was withdrawn by cardiopuncture and collected in heparin tubes. Lungs, spleen, and liver were homogenized and dilutions of the lysates and blood were plated on agar blood plates for determination of CFUs as previously described [24,36].

### 4.7. Histology and Immunohistochemistry

Lung, spleen, and liver were fixed in 10% formaldehyde and embedded in paraffin. Four-micrometer sections of the lung were stained with hematoxylin and eosin (H&E) and scored as described [22]. In short, the following parameters were scored on a scale of 0 (absent), 1 (mild), 2 (moderate), 3 (severe), and 4 (very severe): interstitial damage, vasculitis, peribronchitis, edema, thrombus formation, and pleuritis. Liver necrosis was assessed visually by evaluating the proportion of the liver section area with necrotic tubules. The number of thrombi was counted in the spleen section area. In all experiments, the samples were scored by the same pathologist who was blinded to the experimental groups.

Neutrophil influx was determined by immunohistochemical staining with the Ly-6G monoclonal antibody (mAb; clone 1A8; BioLegend, San Diego, CA, USA). Slides were scanned with the Philips IntelliSite Ultra Fast Scanner 1.6RA (Philips Digital Pathology Solutions, Best, The Netherlands), and TIFF images, spanning the full tissue section, were generated. In these images, Ly-6G positivity and total surface area were measured using ImageJ (version 2006.02.01, U.S. National Institutes of Health, Bethesda, MD, USA); the amount of Ly-6G positivity was expressed as the percentage of the total surface area.

### 4.8. Protein Assays

Human tumor necrosis factor (TNF)-α and interleukin (IL)-6 concentrations were measured using commercially available ELISAs according to the protocol supplied by the manufacturer (R&D Systems, Minneapolis, MN).

Murine TNF-α, IL-6, C-X-C motif ligand (CXCL)1, CXCL2, myeloperoxidase (MPO) and elastase concentrations were determined by ELISA according to the manufacturer’s instructions (R&D Systems, Minneapolis, MN, USA). In plasma, CC chemokine ligand (CCL)2, IL-6, IL-12, TNF-α and interferon (IFN)-γ were determined using a cytometric bead array multiplex assay (BD Biosciences). Alanine aminotransferase (ALT), aspartate amino-transferase (AST) and lactate dehydrogenase (LDH) were measured in plasma using a c702 Roche Diagnostics (Roche Diagnostics BV, Almere, The Netherlands).

### 4.9. Flow Cytometry

For in vitro experiments, cell viability was assessed with the viability dye eFluor^TM^ 780 (Invitrogen). Monocyte purity was verified with mouse anti-human CD14 FITC (clone M5E2) and mouse anti-human CD16 (clone 3G8) for each subject.

For in vivo experiments, cell subsets in blood and BALF (neutrophils CD45+Ly6G+, inflammatory monocytes CD45+Ly6C+CD11b+, alveolar macrophages CD45+CD11c+SiglecF+) were identified by staining with fixable viability dye eFluor 780 (Invitrogen) and the following antibodies: rat anti-mouse CD16/CD32 (clone 93), rat anti-mouse CD45 PE-eFluor610 (clone 30-F11), rat anti-mouse CD11b PE-Cy7 (clone M1/70), rat anti-mouse CD11c PerCP-Cy5.5 (clone HL3), rate anti-mouse Ly6C Alexa fluor 700 (clone AL-21), rat anti-mouse Siglec-F Alexa Fluor 647 (clone E50-2440) (BD Biosciences), and rat anti-mouse Ly6G FITC (clone 1A8) (Miltenyi). Cell counting was performed using internal microsphere counting standard to each sample (CountBright^TM^, Thermofisher). Data were acquired using flow cytometry (FACS CytoFLEX, Beckman Coulter), and data were analyzed using FlowJo software (BD Biosciences).

### 4.10. Statistical Analysis

For in vitro experiments, multiple technical replicates were studied per condition for each subject and cell type. The number of technical replicates depended on the yield of cells and varied between 4 and 8.

Data are expressed as individual data points with median and interquartile range (in vitro experiments) or box-and-whisker plots (in vivo experiments). Data distribution was assessed by the Kolmogorov–Smirnov test, Shapiro–Wilk test and QQ plots. Comparisons between multiple conditions/groups were performed using Friedman test (for paired in vitro data) or Kruskal–Wallis analysis of variance test (in vivo data) and Mann–Whitney U test followed by False Discovery Rate (FDR) corrected multiple comparison. Normally distributed variables were analyzed using a 2-way ANOVA with Student’s *t*-test followed by FDR corrected multiple comparison. Analysis was performed using GraphPad Prism version 8 (Graph-Pad Software, San Diego, CA, USA). Statistical significance is shown as * *p* < 0.05, ** *p* < 0.01, *** *p* < 0.001, and **** *p* < 0.0001.

## 5. Conclusions

In summary, our study provides new insights into the role of Sirt1 in regulating the immune response during bacterial pneumonia and sepsis. SRT1720, a pharmacological activator of Sirt1, reduced inflammation, organ injury and bacterial dissemination in a murine model of pneumosepsis. These findings highlight the potential of Sirt1 activation as a therapeutic strategy to modulate the immune response and improve outcomes in sepsis. Further research is warranted to dissect the mechanism underlying these beneficial effects and to explore the clinical applicability of Sirt1 activators in treating severe infections and preventing sepsis-related complications.

## Figures and Tables

**Table 1:** SRT1720 reduces alveolar cytokine and chemokine release during pneumosepsis.

	24 h		42 h	
	Vehicle	SRT1720	Vehicle	SRT1720
Alveolar IL-6 (pg/mL)	133 (78-223)	37 (31–75) **	49 (39–62)	46 (33–59)
Alveolar TNF-α (pg/mL)	225 (142–580)	45 (71–96) **	153 (108–403)	100 (60–132) *
Alveolar CXCL1 (pg/mL)	287 (264–413)	96 (78–172) ***	278 (174–356)	135 (102–262)
Alveolar CXCL2 (pg/mL)	615 (536–751)	443 (327–501) **	582 (523–705)	511 (466–578) *

Mice were injected intraperitoneally with 20 mg/kg SRT1720 or DMSO (vehicle) before and 24 h after intranasal inoculation with approximately 10^4^ colony-forming units (CFUs) of *Klebsiella pneumoniae*; mice were euthanized 24 and 42 h post-inoculation and bronchoalveolar lavage fluid was obtained for measurement of cytokines and chemokines. Differences were analyzed using the Kruskal–Wallis analysis of variance test and Mann–Whitney U test followed by False Discovery Rate corrected multiple comparison: * *p* < 0.05, ** *p* < 0.01, *** *p* < 0.001 (versus vehicle).

**Table 2:** SRT1720 reduces plasma cytokine levels during pneumosepsis.

	24 h		42 h	
	Vehicle	SRT1720	Vehicle	SRT1720
Plasma IL-6 (pg/mL)	694 (429–1052)	527 (328–1022)	602 (351–1554)	207 (89–364) ***
Plasma TNF-α (pg/mL)	165 (54–375)	84 (49–482)	98 (63–172)	57 (24–103) *
Plasma IFN-γ (pg/mL)	8.9 (2.5–20.6)	2.5 (2.5–19.2)	45.6 (15.8–94.2)	47.6 (20.1–114.7)
Plasma IL-12 (pg/mL)	111 (89–134)	103 (82–157)	233 (98–278)	152 (123–217)

Mice were injected intraperitoneally with 20 mg/kg SRT1720 or DMSO (vehicle) before and 24 h after intranasal inoculation with approximately 10^4^ colony-forming units (CFUs) of *Klebsiella pneumoniae*; mice were euthanized 24 and 42 h post-inoculation and plasma was collected to measure cytokines. Differences were analyzed using the Kruskal–Wallis analysis of variance test and Mann–Whitney U test followed by False Discovery Rate corrected multiple comparison: * *p* < 0.05, *** *p* < 0.001 (versus vehicle).

## Data Availability

Dataset available on request from the authors. Published RNA sequencing are openly available in in the Gene Expression Omnibus of the National Center for Biotechnology Information with accession number GSE160329 35.

## Abbreviations

The following abbreviations are used in this manuscript:

ALT	alanine aminotransferase
AST	aspartate amino-transferase
BALF	bronchoalveolar lavage fluid
CAP	community-acquired pneumonia
CCL	CC chemokine ligand
CFU	colony-forming units
CXCL	C-X-C motif ligand
FDR	false discovery rate
H&E	hematoxylin and eosin
IFN	interferon
LDH	lactate dehydrogenase
NF-κB	nuclear factor-kappa B
PBMCs	peripheral blood mononuclear cells
RPMI	Roswell Park memorial institute
SEAP	secreted alkaline phosphatase
Sirt-1	sirtuin-1
